# Global FDI inflows and outflows in emerging economies Post-COVID-19 era

**DOI:** 10.1186/s43093-022-00167-z

**Published:** 2022-11-08

**Authors:** Omar Al-kasasbeh, Amro Alzghoul, Khaled Alghraibeh

**Affiliations:** Faculty of Business, Amman Arab University, Amman, Jordan

**Keywords:** FDI flows, Emerging economies, Growth, COVID-19

## Abstract

FDI (Foreign Direct Investment) is frequently viewed as a critical measure of a country's economic strength and potential. Consequently, this paper investigates why countries attract FDI by utilizing factors and channels such as vertical or horizontal FDI as well as COVID-19’s impact on FDI flows in emerging economies with data from 1990 to 2020. Models of kinked exponential growth for estimating growth rates and the Andrew and Zivot trend formulations are used to analyze the rise in FDI inflows. The FDI inflow channels are estimated using dynamic panel data analysis, with a generalized method of moments for emerging economies as a whole and an autoregressive distributed lag-pooled mean group for specific countries. The countries studied were India, China, Russia, South Africa, and Brazil. Except for India, where the trend has accelerated, the rest of the nations in the emerging economies category has seen significant or minor declines. Overall, vertical and horizontal factors influence FDI inflows to emerging economies. However, estimations show that vertical and horizontal factors promote FDI inflow into the Russian Federation and India. China's horizontal motivation, on the contrary, is critical. Inflows of FDI into Brazil and South Africa appear to be unrelated. The pandemic scenario affects FDI in Brazil but not in other emerging economies. FDI determinants differ per country. In order to improve their economic situation following the pandemic, developing countries may establish adequate FDI policies to attract FDI.

## Introduction

In emerging countries, Foreign Direct Investment (FDI) has assumed a critical role in capital formation and knowledge transfer. It has been a significant growth booster by giving external resources, new technologies, capacity building, and employment possibilities. The literature indicates that FDI promotes economic growth by easing access to foreign markets and providing capital, foreign exchange, and technology [14]. In addition, they assumed that FDI was injecting domestic investment and innovation to generate economic growth. Countries with low-level equilibrium, characterized by low investment and poor per capita growth as a result of low savings rates, can escape this trap by importing more FDI capital [24]. Besides, FDI has been more steady or less risky compared to other capital flows such as debt flows and portfolio investments [1]. Although FDI presents significant opportunities for the development of emerging countries in a variety of ways, it is severely impacted by global economic volatility. Occasionally, global economic crises impede the global spread of FDI.

Governments all over the globe have taken drastic measures to prevent the spread of COVID-19 [39]. In addition to having a negative impact on the economy, these public health efforts have also had an impact on FDI. The effectiveness of both the health and economic policy initiatives will determine the overall impact on FDI flows [25]. According to the United Nations Conference on Trade and Development (UNCTD), FDI flows witnessed a tremendous rebound in 2020, increasing by 76% to approximately $1.66 trillion, slightly higher than 2019 $928.8 billion surpassing their pre-COVID-19 levels. The COVID-19 pandemic has significantly influenced global FDI flows, which is expected [31]. Consequently, there is a need to conduct more research on the same topic.

According to the World Development Indicators, emerging economies had an annual average growth rate of nearly 5% from 2000 to 2018, compared to 2.8% for the rest of the world over the same time. In the last few decades, the percentage of global Gross domestic product (GDP) generated by emerging economies has almost quadrupled. However, as evidenced by their export vs. import and savings vs. investment discrepancies, emerging economies generally face a capital deficit [3]. Emerging countries cannot fulfill their investment demands due to a lack of capital [19]. As a result, foreign money from industrialized countries must be channeled in the form of FDI to speed up industrialization, reduce unemployment, and create long-term economic development. According to Kose and Ohnsorge [28], emerging market economies saw enormous capital inflows when they were experiencing their first recession in 60 years. Despite the terrible prognosis, one obvious road to recovery is emerging despite the gloom.

FDI is different from other types of capital flows like portfolio investment because it involves long-term and permanent business commitments. Because of this, FDI encourages investors to take a more active part in making decisions [46]. In addition, FDI has long been regarded as a critical building component for developing markets, it has surpassed both government development aid, and portfolio investment flows as the most important source of external funding [41]. Prior to the onset of COVID-19, FDI flows had already begun to slacken due to growing protectionism and other concerns that were eroding investor confidence. Over the last three decades, FDI to emerging economies has increased from about 5% in 2000 to almost 19% in 2020 [49]. FDI inflows take different forms in different countries, primarily due to economic power and infrastructure disparities [21]. Infrastructure, trade openness, market size, inflation rate, human capital, and currency rate, according to the majority of studies, are the most critical determinants affecting FDI inflows to a nation [15], which were all affected by the COVID-19 pandemic.

Because of the pandemic, there is now an additional—and unprecedented—risk in the equation, which has caused business confidence to reach record lows and resulted in an estimated decrease of 42% in global FDI flows [20]. In 2022, The United Nations Conference on Trade and Development (UNCTAD) predicts a 5–10% decline in FDI. Due to pandemic-related concerns, FDI—a critical source of funding for emerging economies—is expected to remain sluggish. Over $100 billion left the area in early 2020, more than three times the volume during the financial crisis. The significant capital flight caused major developing market currencies to depreciate 15%, increasing import costs. 5–10% FDI decline in 2022 [4]. According to UNCTAD, FDI is a critical source of foreign money for a country's economic development, industrialization, and restructuring [26]. By aiding governments in combating the pandemic, assisting their affiliates, and building ties with local businesses, FDI also had a significant role in stabilizing economies during the financial crisis.

The present study investigates FDI influx patterns, causes, and routes, as well as COVID-19's influence on FDI inflow in emerging economies between 1990 (before COVID-19 impacts) and 2020 (during COVID-19 effects). This article makes a threefold contribution. First, it looks at FDI inflows as a proportion of GDP over time. Second, the article applies the knowledge-capital model to find FDI inflow motives. Moreover, the variables affecting vertical and horizontal FDI have been empirically investigated to assess their importance. To identify FDI drivers, the modified K-C model employs various variations of host and source country factors [37]. The G7 nations served as the study's source countries, which can be attributed to G7 nations accounting for the majority of FDI inflows to emerging economies [2]. In addition, the study investigated whether the determinants reacted similarly for all of the emerging economies studied. Finally, the effects of the COVID-19 pandemic on FDI flows have been investigated for the emerging economies nations collectively as well as individually. The current crisis's severity has prompted the investigation of COVID-19's impact on FDI flows. Emerging countries differ from list to list, however in this study, the five most prominent emerging economies are utilized, and two of them are among the top twenty FDI host economies. Emerging economies including. Indonesia, India, Turkey, South Africa, and Brazil are distinguished by rapid economic development and modernization, as well as a rapidly increasing middle-class population, all of which have a significant effect on the regional and global economy.

## Literature review

### Evolution of FDI in emerging economies

Emerging economies have always been of greater interest to foreign investors, particularly those from developed nations. When the Soviet Union collapsed, investors started to focus on emerging economies. This resulted in political and economic adjustments in a number of emerging economies. According to figures published by the International Monetary Fund, the value of FDI from emerging economies accounted for over a quarter of the worldwide stock of foreign direct investment at the end of 2000, or approximately $6.8 trillion [42, 42]. The process of globalization and internationalization of the world's economies have compelled emerging nations to open and liberalize additional sectors, such as transportation and telecommunications that were previously closed to foreign investment. Foreign direct investment decreased by 17% from 2007 to 2008. In light of the global economic crisis of 2009, emerging economies maintained a far stronger position than developed ones. After the recession of 2009, FDI continued to rise, and according to Chollisni et al. [11], the BRICS economies' share of global foreign investment in 2012 reached 20%. Due to the fragility of the economy and the sluggish growth of some economies, FDI has fallen between 2016 and 2018. Even if there is rapid growth in GDP and commerce over that period, foreign investment flows declined by 1.43 trillion dollars, nearly 23%, in 2017 [40].

### The trend and the factors of FDI inflows

There is a lot of conflicting empirical evidence about the trends and drivers of FDI inflows into an economy. Human capital, market size, trade openness, and interest rate have all been identified as significant factors of FDI inflows in Asian emerging nations [29]. FDI inflows to Indonesia are influenced by the new tax treaty and evaluations of the current tax treaty [10]. The characteristics of the local market were significant factors in drawing FDI into the Brazilian economy [16].

Furthermore, while increased productivity in Brazil encourages FDI inflows, productivity growth in the United States discourages them [17]. Market size, interest rate, infrastructural facilities, trade openness, labor cost, gross capital creation, macroeconomic stability, and GDP growth rate have all been identified as critical predictors of FDI inflows in the context of nations that comprise emerging economies [43]. Social, economic, and financial reasons, on the contrary, accounted highly for overall FDI inflows to emerging economies [22].

### FDI inflow motives

Several studies have utilized the knowledge-capital model to investigate the determinants of FDI based on two main FDI motives, i.e., vertical and horizontal. According to Nguyen and Cieslik [36], the most important predictors of FDI inflows to Asian nations were found to be the GDP gap between the source country and the receiver of investments, the distance between two countries exchanging trade or investments, and the costs of trading among the two nations [44]. Cheap labor costs and market potential attract FDI [5]. In contrast, human and physical capital availability and market scope stimulate FDI influx from the Organization for Economic Cooperation and Development (OECD) countries, while investment costs deter inbound FDI [12]. Distance between two countries engaging in trade, the availability of skilled labor, the cost of investments, market size and trade cost, and market size were all crucial determinants in FDI outflows in emerging economies [13]. The availability of trained labor and the size of the market largely impacted the outflow of FDI from the USA [48]. According to previous research, the effect of FDI on the host nation varies depending on the kind of FDI. According to Beugelsdijk et al. [6], horizontal FDI has a greater positive influence on host country economic development than vertical FDI. In Southeast Asian nations, however, vertical and horizontal FDI elevated financing limitations for indigenous firms [8]. From 2002 to 2020, there was a substantial increase in FDI inflows. In parallel with FDI inflows, there has been a significant increase in the number of companies with foreign capital. Since the early 2000s, Turkey has experienced a net FDI inflow of around $209 billion [32].

### How crises affects FDIs

The 2008 financial crisis had a substantial negative impact on FDI in emerging economies [18]. FDI inflows, FDI stocks, and Greenfield FDI operations all suffered considerable losses due to the currency and financial crises [30]. On the contrary, the inflation issue has had minimal influence on FDI activities. During the Asian crisis and recovery period, Moon et al. [33] discovered that both incoming and outbound FDI had an impact on economic growth, resulting in a steady rather than a sudden upturn in Asian economies. There is a scarcity of research on crises' impact on FDI in emerging economies. In the post-2008 global economic crisis, Chattopadhyay et al. [10] found an increase in FDI to India, and Brazil compared to the pre-crisis era, but the financial crisis negatively impacted FDI to South Africa. In contrast, after the global financial crisis of 2008, India and Indonesia were the only nations that had not recovered in terms of FDI inflows and outflows [35]. According to Molano [33], the recovery of emerging economies after the financial crisis was largely dependent on the recovery of the USA and European countries.

### Global FDI and COVID‑19

COVID-19 has seriously hit the entire world economically, socially, and psychologically. COVID-19 increased export and import costs, hence reducing international trade and causing inefficiency [47]. Due to unemployment and the loss of working hours, income decreased. As a result, total aggregate demand had decreased, leading to a decline in output [40, 50]. The primary source of revenue for many nations has been seriously harmed by the pandemic. In addition, global FDI flows fell by one in three to $1 trillion in 2020, the lowest level since the global financial crisis. COVID-19 has harmed the most productive forms of investment, such as greenfield investment in infrastructural projects and industries, particularly in developing nations. This suggests that international production, the engine of world economic growth and development, has been significantly impacted [23]. The destructive effects of the pandemic on the trajectory of FDI flow vary between developing and developed nations. COVID-19 has had a negative effect on FDI inflows, particularly for investments in global value chain (GVC)-intensive and tourism in developing and transition economies [45]. In contrast, greenfeld FDI inflows had had a decline since 2018. The epidemic accelerated this decline, particularly in doping nations. Africa has been hit the hardest region in greenfield FDI flows, with a 65% drop. Latin America and the Caribbean is in second position with a 51% drop, and Asia is in third [27]. The COVID-19 outbreak has had the same effect on FDI flows around the world. It was expected that the health crisis would have a lot of bad effects on developing countries. Most of the FDI that goes to developing countries comes from the primary sector. This means that the negative effects are not all the same. In other words, FDI in these countries is mostly focused on commodities, whose prices have dropped because of a lack of demand caused by pandemic restrictions [7, 38].

Previous research on FDI has been evaluated to identify results and conclusions that have been overlooked. A review of the prior literature reveals several studies on emerging economies. However, the majority of them have solely looked at FDI factors. Few studies have examined FDI inflows and the impact of crises on FDI in emerging economies. Furthermore, few studies employ the K-C model to investigate FDI drivers and motives (vertical or horizontal) in emerging economies. Moreover, since the COVID-19 problem is a recent occurrence, there has been no research on its influence on FDI inflows. Furthermore, in the context of the emerging economies, this issue has not been addressed. Furthermore, to our knowledge, determinants defining the kind of FDI, i.e., vertical or horizontal, are yet to be investigated in the emerging economies framework.

## Data and methodology

### Theoretical background and data sources

The current research is based on secondary data from time series sources, specifically the OECD Economic Outlook, the World Bank database, and the PENN World Table (PWT). For our research purposes, we used data series that was monitored yearly from 1990 to 2020. COVID-19's influence on FDI inflows was also studied using quarterly data. The research period is solely determined by the accessibility of a current data series that includes all variables. For uniformity, all data are expressed in million US dollars. The descriptions of the relevant variables and data sources are listed in Table 1.

**Table 1:** Data sources

Data	Sources
Real GDP	OECD Stat
FDI inflows	OECD Stat
Capital stock	Penn World Data
Control of Corruption Index	World Bank database
Exports	OECD database
Human capital index	Penn World Data
Distance	Google distance calculator
Import	OECD database
People engaged	Penn World Data

### The structural break test

Unknown (endogenous) breakpoint tests like Quandt-structural Andrews's breakpoint test were meant to identify a sudden or abrupt rise or reduction in the movement of time series data. Later, in order to further corroborate the breakpoints identified by the Quandt-Andrews test, the Chow Breakpoint Test was performed, as depicted in Table 2, which reveals that both the Quandt-Andrews and the Chow tests used to estimate the results show that there is a single large split in the time—series data for each nation, although the break occurs in different periods. Structured vulnerabilities must be identified and quantified in order to forecast FDI growth patterns.

**Table 2:** Unit root test statistics and structural break

Structural break test		Quandt-Andrews unknown breakpoint test		Chow breakpoint test	Stationarity test
Countries	Break point	Maximum LR F-statistic	Ave LR F-Statistic	Log-Likelihood Ratio	Phillips—Perron's Test Statistic
India	2006	19.0973*	4.9226*	27.1107*	− 1.8570***
Turkey	2003	32.0612*	11.026*	33. 5229*	− 2.2107***
Indonesia	1999	17.5239*	5.0631*	26.0239*	− 1.7346***
South Africa	2004	4.6461***	25,257**	9.1671**	(− 4.7751) *
Brazil	2002	23.582*	6.3072*	31.0388*	− 1.9240***

^***^, **, *Represent significant values at 1%, 5%, and 10% levels, respectively

### The conditional-unconditional growth rate and stationarity tests

The Phillips–Perron nonparametric integration test was used to determine the stationarity of the time series. According to Table 2, the series is stable in South Africa, as predicted by the Phillips–Perron test, as previously stated. The series of FDI as a proportion of growth domestic product, on the other hand, is shown to be nonstationary in the remaining four nations. For the purpose of dynamic panel data analysis, the panel unit root test developed by Levin-Lin-Chu (2002) is utilized to determine whether the variables used for the analysis are stationary.

When dealing with a stationary series, the classic trend analysis is carried out by calculating the kinked exponential trend:

1 $$\ln y_{t} = \alpha + \beta_{1} d_{1} t + \beta_{2} d_{2} t + \varepsilon_{t} .$$

We apply the kinked exponential (deterministic) trend analysis if the series is not stationary. By multiplying *β*_1_ and *β*_2_ by 100, the trend equation calculates the yearly growth rate in y for each subperiod. Subperiod trend changes were assessed using the Andrews and Zivot trend equation: The first subperiod is 0 and the second is 1; the estimated value of β_1_ represents the change in y's level over subperiods, whereas the estimated value of β_2_ represents the trend in y's level over subperiods.

## Main estimation

### Dynamic panel analysis

The prevalence of heterogeneity is evident since the research deals with many variables impacting FDI inflow across nations and time. Panel Regression Analysis captures this variability between units by allowing for specific fluctuations in specific countries across time. Fixed-effects, random-effects, and Gaussian mixture models (GMM) are strategies used in Panel Data Regression Analysis. To choose between random-effects model and fixed-effects model, the Hausman specification test is used. Heterogeneity among countries may also be managed by using Dynamic Panel Evaluation. The endogeneity issue in regressors is evident in this regression, as is the serial correlation problem. Due to the delayed nature of the explanatory variables, we utilized GMM to estimate the Panel Analysis's parameters. GMM can solve the endogeneity problem through proper modeling may help to avoid it. The autoregressive model was estimated using Pooled Mean Group estimation, including I(0) and I(1) variables. The typical model formulation for testing hypotheses about the drivers of FDI flows is as follows:

2 $$Y_{t} = {\mathfrak{a}} + \gamma Y_{t - 1} + \beta_{1} X_{t} + \varepsilon_{t} ,$$

where *Y*_*t*-1_ is the lagged dependent variable, *Y*_*t*_ is the dependent variable, whereas *X*_*t*_ is a collection of explanatory factors, such as Gross domestic product Difference **(**GDP_dff_), Gross domestic product Sum (GDPs), physical capital, trade openness, human capital, corruption control, and distance.

## Empirical results and discussion

### Growth

For both Brazil (32.6%) and Turkey (12.8%), Table 3 shows a significant increase in FDI as a proportion of gross domestic product over time, nevertheless for Brazil, we also find a considerable reduction, both in terms of absolute value and in terms of trend (77.6% and 31.6%). In contrast, India (77.9%) and Turkey (68.2%) demonstrate significant growth at the national level; they also show a declining trend in the second subperiod, with 7.23% and 30.5%, respectively. The growth rates of Indonesia (42.3%) and South Africa (33.6

**Table 3:** Conditional and unconditional FDI growth estimates

		*B*	*B*_1_	*B*_2_
Indonesia	Coefficient t-Statistic		0.423 5.428***	− 0.0802 − 5.243***
Brazil	Coefficient t-Statistic	0.326 2.322**	− 0.776 − 1.838*	− 0.316 − 2.232
India	Coefficient t-Statistic	0.046 1.214	0.779 2.268***	− 0.0723 − 1.788*
South Africa	Coefficient t-Statistic		0.336 4.062***	− 0.027 − 0.803
Turkey	Coefficient t-Statistic	0.128 2.082**	0.682 1.709***	− 0.305 − 2.973**

Up to lag 4, the t-Statistic^#^ is based on the lowest AIC where it is lag 4 for Brazil and Turkey and lag 2 for India

%) in the first subperiod are statistically significant. In comparison, Indonesia (8%) and South Africa (2.7%) substantially reduced during the second subperiod.

### Motives of FDI

Table 4 demonstrates that all explanatory variables are stable, excluding variable control of corruption, which is not included in the model due to its nonstationary nature. "1(0)" represents stationarity at level, at the trend of the underlying panel by using the Levin-Lin-Chu test for stationarity. The dependent variable is FDI inflow.LI, while the independent variables are GDPs, physical capital, GDP_dff_, human capital, trade openness, distance, and control of corruption.

**Table 4:** Testing for stationarity and generalized method of moments results

Variables	Levin-Lin-Chu Statistic for stationary	Fixed effect		GMM	
		Coefficient	*t*	Coefficient	*t*
FDI inflow LL			0	0.237436	2.06**
GDP_s_	(− 4.5904***)	1.9678	4.13**	1.4279	4.17***
Trade openness	(− 5.8044***)	0.8804	− 2.97*	2.3711	3.02
GDP_dff_	(− 8.3116***)	− 0.0124	− 0.04	− 0.1223	− 1.97*
Human Capital	(− 4.9129**)	1.04189	− 3.07*	− 0.9124	− 2.01**
Distance	(− 3.6179***)	0.9716	3.63*	0.9859	5.73***
Physical Capital	(− 2.1862**)	− 1.5178	− 3.07*	− 0.9123	2.35**
Control of corruption	(− 1.3051)				

The Hausman test for the panel data reveals that the results are statistically significant, leading to the adoption of the fixed-effects model. Table 4 compares the estimated results using the fixed-effects model and generalized method of moment’s estimators. Fixed-effect models find statistical significance in the estimates of trade openness, physical capital, distance, and GDPs. The estimates of explanatory variables, as well as the expected sign, are substantially significant in generalized multinomial models, favoring FDI inflows from both horizontal and vertical angles. The robust GMM estimates (i.e., those that are not affected by the endogeneity problem) produced using dynamic panel data regression analysis are the subject of this paper.

The horizontal motive forecasts a positive relationship between the GDPs of the host country and the amount of FDI inflows. In addition, we observe a statistically significant positive coefficient of the aggregate economic size of each of the nations in emerging economies and the G7 countries, indicating the existence of a horizontal motive for FDI inflows. As depicted in Table 4, inbound FDI into the host nations is negatively associated with differences in country size, indicating a horizontal desire to access the host market. Given that the GDP_dff_ is negative and statistically significant, the variable is being used for cost reduction, as well as is vertical. The human capital proportion (rather than the difference between physical and human capital) is expected to show a significant positive coefficient, indicating that more FDI will be directed to the host country with a more developed human resource. When it comes to the regressors, we get some surprising outcomes. The trade openness variable establishes a statistically significant positive relationship with FDI inflow. Contrary to the common assumption, the distance variable establishes a statistically significant positive relationship with FDI inflow, these anomalous empirical findings could have been avoided by increasing the size of the sample or refining the variables under consideration, even though the current study acknowledges the limitations of not being able to collect categorized data for a prolonged period of time.

Table 5 depicts the estimates of the Pooled Mean Group (PMG) for each nation studied. While assessing country-wise an autoregressive distributed lag using the Pooled Mean Group, there is no concave function due to the limited number of units (countries) compared to the length of period considered in estimating the function by PMG (i.e., number of years). In order to get a concave function, PMG is run using just explanatory variables (i.e., the GDPs and GDP_dff_). Based on the results, the coefficient for GDPs for India, Indonesia, and Turkey is positively statistically significant, whereas the coefficient GDP_dff_ for Indonesia is negatively statistically significant. The coefficient of GDPs for Turkey and India is positive and statistically significant. Therefore, it has been proved empirically that both vertical and horizontal motives are important drivers for FDI inflows to Turkey and India. The motives underlying FDIs in Brazil and South Africa are insignificant, as shown in Table 5.

**Table 5:** PMG estimates

		Coefficient	t
Indonesia	*GDPs* ${GDP}_{dff}$	18.804 0. 803	1.012*** − 3.223***
Brazil	${GDP}_{s}$ ${GDP}_{dff}$	31.776 0.875	− 1.31 1.23
India	${GDP}_{s}$ ${GDP}_{dff}$	26.479 2.268*	1.723** 2.88**
South Africa	${GDP}_{s}$ ${GDP}_{dff}$	− 52.934 24.437	− 1.27 1.42
Turkey	${GDP}_{s}$ ${GDP}_{dff}$	29.804 23.437	2. 63 1.36

The dependent variable is FDI inflow, and the independent variables are represented as GDPs and GDP_dff_. A few values of S.E. and t-Statistics are not generated due to their substantial and small values

### The impacts of COVID-19 on foreign direct investment flows

In order to evaluate the impacts that COVID-19 had on FDIs to emerging economies, quarterly data were collected from 2019 to 2020. The study utilized GDPs and GDP_dff_ as the sole independent variables since these were the only variables available for the quarterly data. An evaluation of all dependent variables' stationarity was made and is shown below (Table 6).

**Table 6:** Testing for Stationarity of Exploratory Variables

Variables	Levin-Chutest for stationary	Random effect		GMM	
		Coefficient	*t*	Coefficient	*t*
FDI inflow LL				− 0.158683	(− 1.14)
GDPs	(− 2. 2582***)	3.5449	1.17	8.299980	1.91**
GDP_dff_	(− 2.2301***)	− 0.2456	− 0.43	− 0.484410	0.71
COVID period*	(− 1.7662***)	− 0.2624	− 0.58	− 0.481312	0.82

All variables are found to be 1(0) at 1% level

Based on PMG calculations, Table 7 reveals that COVID-19's time dummy is insignificant for all emerging economies. It is far too early to assess the effect of such a small sample size. In the midst of the pandemic, nonetheless, Brazil's vertical strategy of market access is proving effective, as seen by the country's notable negative GDPs and positive significant GDP_dff_.

**Table 7:** Pooled mean group estimates

		Coefficient	*t*
Indonesia	${GDP}_{s}$ ${GDP}_{dff}$ Time dummy	− 27.110 8.533 − 0.026	(− 0.04) 0.02 0.01
Brazil	${GDP}_{s}$ ${GDP}_{dff}$ Time dummy	− 83.708 34.899 − 0.722	(6.31*) 6.14* (− 1.27)
India	${GDP}_{s}$ ${GDP}_{dff}$ Time dummy	− 19.654 2.668 0.459	– – 0.05
South Africa	${GDP}_{s}$ ${GDP}_{dff}$ Time dummy	− 16.034 6.466 − 0.007	(− 0.02) 0.03 0.00
Turkey	${GDP}_{s}$ ${GDP}_{dff}$ Time dummy	13.675 − 0.274 0.709	0. 11 − 0.05 0.05

^**#**^**-** All the estimated results, which were small for India, did not generate. Due to their substantial and small values, a few S.E. and t-Statistic values are not generated

## Conclusion and policy recommendations

The research attempted to interpret the growth patterns in FDI inflows, the drivers of FDI, determinants of FDI, and channels of inflow of FDIs, as well as how foreign COVID-19 impacted direct investment inflows in emerging markets between 1990 (before COVID-19 effects) and 2020 (during COVID-19 effects) when the pandemic's effects mainly were felt. The current study was necessary since a FDI is a critical component of a free and open international economic system, as well as a primary driver of growth and which had been badly hurt by the COVID-19 pandemic. The article contributed in three ways. First, it calculated FDI inflows as a proportion of GDP throughout the time period under study. Second, the article utilized the knowledge-capital model to determine FDI inflow motives. In addition, the factors influencing FDI were evaluated together to estimate their relative relevance. G7 nations were utilized as the source of FDI to the emerging economies. Finally, the impacts that COVID-19 had on FDIs to the emerging economies for individual and collective countries. The research focused on India, Turkey, Indonesia, South Africa, and Brazil as the emerging economies. The study used secondary data from time series sources such as the OECD Economic Outlook, World Bank database, and PENN World Table. Our analysis utilized annual data from 1990 to 2020.

While India have shown consistent growth, it is different for Indonesia and Brazil, with South Africa and Turkey remaining stagnant. The study used panel data analysis using generalized method of moments for the emerging economies as a whole and an autoregressive distributed lag for individual nations to estimate FDI inflow channels. According to empirical estimates, vertical and horizontal motives influence FDI in emerging economies. Although country-by-country assessments demonstrate that both vertical and horizontal reasons drive FDIs into Turkey India, for Indonesia, the horizontal motive alone influences that No FDI motive appears significant for Brazil or South Africa. A primary motivation for conducting this research is to examine the impact of the COVID-19 pandemic on FDI into emerging economies. The time when the pandemic occurred was used as a dummy variable along with other control factors. Based on the findings, while the epidemic had little effect on luring FDIs to other emerging economies, it significantly impacted Brazil.

The study's overall policy recommendation for the emerging economies is to implement more robust reforms in order to attract FDI. Emerging economies should allow FDIs to be integrated more deeply in their economies. Developing quality certification systems (often required to work with foreign firms), improving digital infrastructure (allowing firms to work remotely throughout global value chains and reach foreign markets), and designing export processing zones (EPZs) have all been shown to be effective.

Like other empirical studies in this field, the current study has its own set of limitations. Its conclusions should not be applied with certainty because they are heavily reliant on the currently available data set. More of these studies are needed in order to generalize the results.

## Data Availability

Not applicable.

## Abbreviations

EPZs: Export processing zones
FDI: Foreign direct investment
GDP: Gross domestic product
GDP_dff_: GDP difference
GDPs: GDP Sum
GMM: Gaussian mixture models
OECD: The organization for economic cooperation and development
(PWT): The PENN World Table
UNCTAD: The United Nations Conference on Trade and Development
